# Prognostic Significance of Arterial Lactate Levels at Weaning from Postcardiotomy Venoarterial Extracorporeal Membrane Oxygenation

**DOI:** 10.3390/jcm8122218

**Published:** 2019-12-15

**Authors:** Fausto Biancari, Antonio Fiore, Kristján Jónsson, Giuseppe Gatti, Svante Zipfel, Vito G. Ruggieri, Andrea Perrotti, Karl Bounader, Antonio Loforte, Andrea Lechiancole, Diyar Saeed, Artur Lichtenberg, Marek Pol, Cristiano Spadaccio, Matteo Pettinari, Krister Mogianos, Khalid Alkhamees, Giovanni Mariscalco, Zein El Dean, Nicla Settembre, Henryk Welp, Angelo M. Dell’Aquila, Thomas Fux, Tatu Juvonen, Magnus Dalén

**Affiliations:** 1 Heart Center, Turku University Hospital and Department of Surgery, University of Turku, 20521 Turku, Finland; 2 Research Unit of Surgery, Anesthesiology and Critical Care, University of Oulu, 90570 Oulu, Finland; tatu.juvonen@hus.fi; 3 Department of Cardiothoracic Surgery, Henri Mondor University Hospital, AP-HP, Paris-Est University, 94000 Créteil, France; fioreant7@yahoo.com; 4 Department of Cardiac Surgery, Sahlgrenska University Hospital, 41685 Gothenburg, Sweden; kristjan.jonsson@vgregion.se; 5 Division of Cardiac Surgery, Ospedali Riuniti, 34121 Trieste, Italy; gius.gatti@gmail.com; 6 Hamburg University Heart Center, 20246 Hamburg, Germany; s.zipfel@uke.de; 7 Division of Cardiothoracic and Vascular Surgery, Robert Debré University Hospital, 51100 Reims, France; vgruggieri@chu-reims.fr; 8 Department of Thoracic and Cardio-Vascular Surgery, University Hospital Jean Minjoz, 25000 Besançon, France; a.perrotti@hotmail.it; 9 Division of Cardiothoracic and Vascular Surgery, Pontchaillou University Hospital, 35000 Rennes, France; karl.bounader@hotmail.com; 10 Department of Cardiothoracic, Transplantation and Vascular Surgery, S. Orsola Hospital, University of Bologna, 40138 Bologna, Italy; antonino.loforte@aosp.bo.it; 11 Cardiothoracic Department, University Hospital of Udine, 33100 Udine, Italy; andrea.lechiancole@asuiud.sanita.fvg.it; 12 Cardiovascular Surgery, University Hospital of Duesseldorf, 40225 Dusseldorf, Germany; diyar.saeed@helios-gesundheit.de (D.S.); artur.lichtenberg@med.uni-duesseldorf.de (A.L.); 13 Institute of Clinical and Experimental Medicine, 14021 Prague, Czech Republic; marek.pol@ikem.cz; 14 Department of Cardiothoracic Surgery, Golden Jubilee National Hospital, Glasgow G814DY, UK; cristianospadaccio@gmail.com; 15 Department of Cardiovascular Surgery, Ziekenhuis Oost-Limburg, 3600 Genk, Belgium; matteo.pettinari@zol.be; 16 Department of Cardiothoracic Surgery, University of Lund, 22184 Lund, Sweden; k.mogianos@gmail.com; 17 Prince Sultan Cardiac Center, Al Hassa 31982, Saudi Arabia; kalkhamees@hotmail.com; 18 Department of Cardiac Surgery, Glenfield Hospital, University Hospitals of Leicester, Leicester LE39QP, UK; Giovannimariscalco@yahoo.it (G.M.); zein.eldean@hotmail.co.uk (Z.E.D.); 19 Department of Vascular Surgery, Nancy University Hospital, University of Lorraine, 54000 Nancy, France; nicla.settembre@yahoo.com; 20 Department of Cardiothoracic Surgery, Münster University Hospital, 48149 Münster, Germany; henryk.welp@ukmuenster.de (H.W.); angelo.dellaquila@ukmuenster.de (A.M.D.); 21 Department of Molecular Medicine and Surgery, Department of Cardiac Surgery, Karolinska Institutet, Karolinska University Hospital, 171777 Stockholm, Sweden; thomas.fux@karolinska.se (T.F.); magnus.dalen@karolinska.se (M.D.); 22 Heart and Lung Center, Helsinki University Hospital, 00290 Helsinki, Finland

**Keywords:** extracorporeal membrane oxygenation, cardiac surgery, postcardiotomy, venoarterial, ECMO, VA-ECMO

## Abstract

Background: The outcome after weaning from postcardiotomy venoarterial extracorporeal membrane oxygenation (VA-ECMO) is poor. In this study, we investigated the prognostic impact of arterial lactate levels at the time of weaning from postcardiotomy VA. Methods: This analysis included 338 patients from the multicenter PC-ECMO registry with available data on arterial lactate levels at weaning from VA-ECMO. Results: Arterial lactate levels at weaning from VA-ECMO (adjusted OR 1.426, 95%CI 1.157–1.758) was an independent predictor of hospital mortality, and its best cutoff values was 1.6 mmol/L (<1.6 mmol/L, 26.2% vs. ≥ 1.6 mmol/L, 45.0%; adjusted OR 2.489, 95%CI 1.374–4.505). When 261 patients with arterial lactate at VA-ECMO weaning ≤2.0 mmol/L were analyzed, a cutoff of arterial lactate of 1.4 mmol/L for prediction of hospital mortality was identified (<1.4 mmol/L, 24.2% vs. ≥1.4 mmol/L, 38.5%, *p* = 0.014). Among 87 propensity score-matched pairs, hospital mortality was significantly higher in patients with arterial lactate ≥1.4 mmol/L (39.1% vs. 23.0%, *p* = 0.029) compared to those with lower arterial lactate. Conclusions: Increased arterial lactate levels at the time of weaning from postcardiotomy VA-ECMO increases significantly the risk of hospital mortality. Arterial lactate may be useful in guiding optimal timing of VA-ECMO weaning.

## 1. Introduction

Venoarterial extracorporeal membrane oxygenation (VA-ECMO) is an effective salvage therapy for postcardiotomy cardiogenic shock refractory to inotropes and intra-aortic balloon pump support [1]. Recent pooled results demonstrated that one third of patients survived to discharge using postcardiotomy VA-ECMO, although 23% of patients died early after weaning from postcardiotomy VA-ECMO [2]. The causes underlying such a high postweaning mortality have not been thoroughly investigated. The identification of risk factors underlying failure to recover after weaning from postcardiotomy VA-ECMO are of clinical importance to establish a protocol for discontinuation of this therapy in patients with unrecoverable end-organ failure as well as to develop strategies to improve the early outcome of postcardiotomy VA-ECMO. Increased levels of arterial lactate before and during ECMO have been shown to predict the outcome of these patients [2,3,4,5,6,7]. In this study, we investigated whether arterial lactate levels at the time of weaning from VA-ECMO is a prognostic factor for weaned patients and could be useful in the decision-making process of the timing of VA-ECMO discontinuation.

## 2. Methods

### 2.1. Study Population

The PC-ECMO registry is a multicenter retrospective study that gathered data on consecutive patients who underwent VA-ECMO after adult cardiac surgery at 19 cardiac surgery centers in Belgium, Czech Republic, Finland, France, Italy, Germany, Saudi Arabia, Sweden, and the United Kingdom, from January 2010 to March 2018. The study is registered in Clinicaltrials.gov (Identifier: NCT03508505) and the main results of this series are reported elsewhere [7]. The institutional review board or the regional ethics review board of each participating center approved this study. Data was collected retrospectively into a dedicated access datasheet with pre-specified variables and underwent checking of its quality.

Consecutive patients aged over 18 years who required mechanical circulatory support with VA-ECMO for cardiopulmonary failure occurring during the index hospitalization after cardiac surgery, i.e., surgery on the coronary arteries, heart valves, ascending aorta/aortic arch and/or ventricular wall and septum, grown-up congenital heart diseases, and chronic thromboembolic pulmonary hypertension, were included in this registry. Cardiopulmonary failure in these patients was considered not responsive to inotropes and/or intra-aortic balloon pump. Patients who received any ECMO therapy before cardiac surgery or VA-ECMO after heart transplantation or implantation of a ventricular assist device were excluded from this registry. Furthermore, patients who underwent heart transplantation or implantation of a ventricular assist device while on VA-ECMO were excluded from the present analysis. For the purpose of this study, we included only patients who were considered safely weaned from VA-ECMO per treatment protocol and with available data on arterial lactate levels immediately before weaning from VA-ECMO.

### 2.2. Outcomes

The outcome measures of the present study were hospital mortality after weaning from VA-ECMO, i.e., all-cause death during the index hospitalization, and 30-day mortality. Specific causes of death were not considered as outcome measures because most often multiple conditions led to early death after weaning from VA-ECMO.

### 2.3. Statistical Methodology

Statistical analyses were performed using SPSS v. 25.0 (IBM Corporation, Armonk, NY, USA) and Stata v. 15.1 (StataCorp LLC, College Station, TX, USA) statistical software. Continuous variables are reported as mean and standard deviation, and categorical variables as counts and percentages. Risk estimates are reported as odds ratio (OR) and hazard ratio (HR) with their 95% confidence interval (CI). Arterial lactate clearance was estimated as the difference of the pre-VAECMO arterial lactate level and the VA-ECMO weaning arterial lactate level. The arterial lactate clearance/duration of the VA-ECMO ratio was also considered as a covariate of interest. The Mann–Whitney *U*, chi-square, the Fisher’s exact, linear-by-linear association tests, and receiver operating characteristics curve (ROC) analysis with estimation of the area under the curve (AUC) were used for univariate analysis. The Youden’s test was used to identify the best cutoff value of arterial lactate at weaning from VA-ECMO in predicting hospital death. The DeLong’s test was used for comparative analyses of ROC curves. Multilevel mixed-effects logistic regression was employed to identify independent predictors of hospital mortality avoiding bias related to any interinstitutional difference. In fact, multilevel models provide a valuable way to identify independent predictors of poor outcome taking into account interinstitutional differences in terms of the referral pathway, treatment strategy, and institutional performance. Regression models included multiple risk factors preceding the initiation of VA-ECMO, with *p* < 0.1 for hospital mortality in the univariate analysis (Table 1). Furthermore, the impact of arterial lactate at weaning from VA-ECMO on hospital mortality was adjusted for the PC-ECMO score [7]. Once pre-VA-ECMO arterial lactate was dichotomized, 1-to-1 propensity score matching analysis was performed to reduce the effect of confounding covariates using the psmatch2 Stata module with a caliper width of 0.2 of the standard deviation of the logit, i.e., 0.1. A propensity score was estimated with non-parsimonious logistic regression, including all covariates listed in Table 2, except the type of surgery and arterial lactate and pH levels at the time of VA-ECMO weaning. Standardized differences lower than 0.10 were considered as an adequate balance between the matched cohorts. The McNemar test was used to assess the difference in all-cause hospital mortality in the propensity score-matched pairs. Mortality at 30 days after weaning from VA-ECMO was evaluated also by the Kaplan–Meier method with the log-rank test and the Cox proportional hazards method. A *p* < 0.05 was set for statistical significance.

## 3. Results

### 3.1. Characteristics of the Study Cohort

The PC-ECMO registry included 781 consecutive patients and hospital mortality was 64.3%. Mortality on VA-ECMO occurred in 362 patients (46.4%). Among 419 patients who were believed to be safely weaned from VA-ECMO, 140 patients (33.4%) died during the index hospital stay. Data on the arterial lactate level before and at weaning from VA-ECMO was available in 355 patients. Seventeen patients underwent heart transplantation or implantation of a ventricular assist device from VA-ECMO and were excluded from the present analysis. Overall, 338 patients were the subjects of this study and their baseline characteristics, operative data, and VA-ECMO-related data are summarized in Table 1. In these patients, the mean VA-ECMO therapy duration was 8.1 ± 6.3 days (median 6.0 days, interquartile range 7.0 days, range 0.5–39.0 days, 5 lowest outliers 0.5–0.8 days; 5 highest outliers 31.0–39.0 days).

### 3.2. Predictive Performance of Arterial Lactate at VA-ECMO Weaning

The AUC of pre-VA-ECMO arterial lactate was 0.541 (95%CI 0.476–0.606) and that of arterial lactate at weaning from VA-ECMO was 0.629 (95%CI 0.567–0.691) (DeLong test, *p* = 0.032). Arterial lactate clearance (AUC 0.521, 95%CI 0.454–0.588) and arterial lactate clearance/duration of VA-ECMO ratio (AUC 0.549, 95%CI 0.483–0.615) were not associated with increased hospital mortality.

Mixed-effects logistic regression showed that arterial lactate at weaning from VA-ECMO (*p* = 0.001, adjusted OR 1.426, 95%CI 1.157–1.758; Likelihood-ratio test for model vs. logistic regression, *p* < 0.0001) was an independent predictor of hospital mortality when adjusted for risk factors with *p* < 0.1 in the univariate analysis (Table 1). Similarly, arterial lactate at weaning from VA-ECMO was predictive of hospital mortality (adjusted OR 1.341, 95%CI 1.100–1.635) when adjusted for the PC-ECMO score.

**Figure 1:**
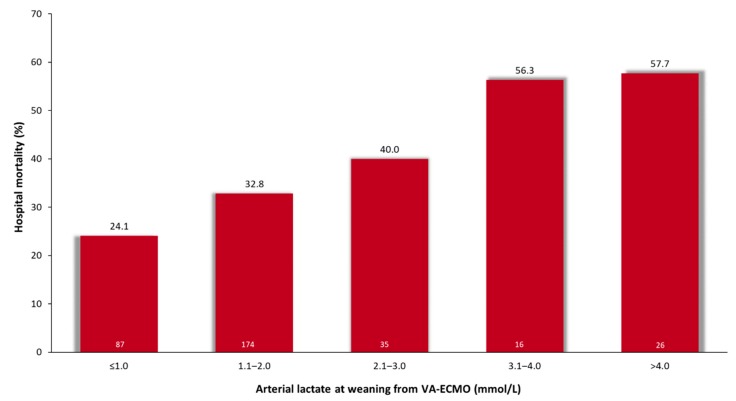
Hospital mortality rates according to increasing arterial lactate levels at the time of weaning from postcardiotomy extracorporeal membrane oxygenation (linear-by-linear association test, *p* < 0.0001). Number of patients in each group are reported at the bottom of bars.

The crude rates of hospital mortality along increasing arterial lactate levels at weaning from VA-ECMO are summarized in Figure 1 (linear-by-linear association test, *p* < 0.0001). The Youden’s test identified a cutoff of arterial lactate of 1.6 mmol/L for the prediction of hospital mortality (arterial lactate <1.6 mmol/L 26.2% vs. arterial lactate ≥1.6 mmol/L 45.0%, *p* < 0.0001, sensitivity 57%, specificity 65%). When adjusted for participating centers and risk factors with *p* < 0.1 in the univariate analysis, arterial lactate at weaning from VA-ECMO ≥1.6 mmol/L was still predictive of hospital mortality (adjusted OR 2.489, 95%CI 1.374–4.505). Similarly, arterial lactate at weaning from VA-ECMO ≥1.6 mmol/L was predictive of hospital mortality (adjusted OR 2.094, 95%CI 1.201–3.651) when adjusted for the PC-ECMO score. Thirty-day mortality after weaning from VA-ECMO was 21.0% in patients with arterial lactate <1.6 mmol/L and 41.1% in those with arterial lactate ≥1.6 mmol/L (log-rank test, *p* < 0.0001; adjusted HR 2.245, 95%CI 1.416–3.560).

### 3.3. Predictive Performance of Mild Hyperlactatemia at VA-ECMO Weaning

The overall study population included a number of patients with arterial lactate at VA-ECMO weaning >2.0 mmol/L. In order to avoid bias of excessively high arterial lactate levels, we assessed the prognostic performance of arterial lactate levels in the subgroup with mild hyperlactatemia as defined by arterial lactate at VA-ECMO weaning ≤2.0 mmol/L (261 patients). The hospital mortality of this subgroup of patients was 30.2%.

**Figure 2:**
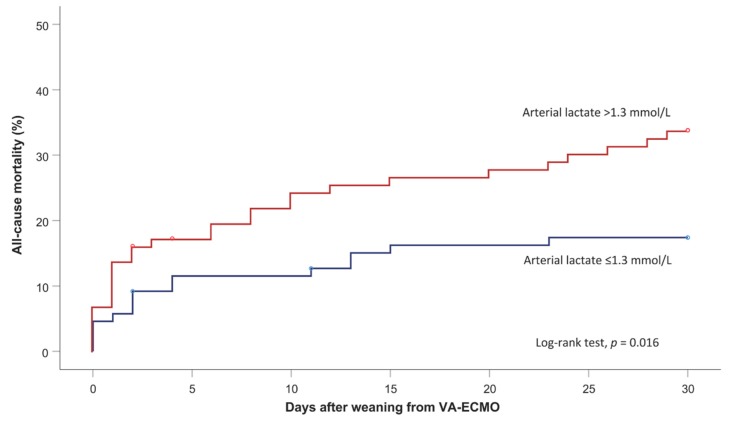
Kaplan–Meier estimates of 30-day all-cause mortality in propensity score matched pairs with arterial lactate levels <1.4 mmol/L versus 1.4 to 2.0 mmol/L at the time of weaning from postcardiotomy venoarterial extracorporeal membrane oxygenation (Log-rank test, *p* = 0.016).

The AUC of pre-VA-ECMO arterial lactate was 0.529 (95%CI 0.452–0.606) and that of arterial lactate at weaning from VA-ECMO was 0.578 (95%CI 0.504–0.652) (DeLong test, *p* = 0.325). The Youden’s test identified a cutoff of arterial lactate of 1.4 mmol/L for the prediction of hospital mortality (arterial lactate <1.4 mmol/L, 24.2% vs. arterial lactate ≥1.4 mmol/L, 38.5%, *p* = 0.014, sensitivity 51%, specificity 65%). Propensity score matching resulted in 87 pairs with balanced baseline characteristics and duration of VA-ECMO (Table 2). Hospital mortality was significantly higher in patients with arterial lactate ≥1.4 mmol/L (34 patients, 39.1% vs. 20 patients, 23.0%, McNemar’s test, *p* = 0.029) compared to those with arterial lactate <1.4 mmol/L. These findings were confirmed also when arterial lactate ≥1.4 mmol/L was adjusted for postoperative renal replacement (OR 1.846, 95%CI 1.068–3.193). Among propensity score-matched pairs, 30-day mortality after weaning from VA-ECMO was 17.4% in patients with arterial lactate at VA-ECMO weaning <1.4 mmol/L and 33.8% in those with arterial lactate ≥1.4 mmol/L (Log-rank test, *p* = 0.016; HR 2.100, 95%CI 1.126–3.919) (Figure 2).

## 4. Discussion

This study demonstrated that there is a substantial hospital mortality after weaning from postcardiotomy VA-ECMO and most of these patients die during the first five days after discontinuation of VA-ECMO therapy (Figure 2). In the absence of a standardized VA-ECMO weaning protocol [8], the present results suggest that treatment strategies at the time of discontinuation of this salvage therapy should be improved. In particular, there is a need for objective parameters indicating when and how to intervene to improve the cardiopulmonary and metabolic status of these critically ill patients and thereby to optimize the timing of weaning from VA-ECMO. In this setting, arterial lactate is an independent predictor of early mortality and may potentially guide the decision-making process before discontinuation of mechanical circulatory support. Importantly, analysis of the subset of patients with arterial lactate ≤2.0 mmol/L at the time of discontinuation of VA-ECMO showed that even a mild increase of lactate, i.e., ≥1.4 mmol/L, is associated with a two-fold risk of early mortality. Such a risk was observed in propensity score-matched pairs with similar duration of VA-ECMO (Table 2). We speculate that patients with mild hyperlactatemia after VA-ECMO therapy, in the absence of irreversible end-organ injury, may need prolonged mechanical circulatory support and treatment of underlying causes of hyperlactatemia. In the absence of suboptimal oxygen delivery and severe renal failure, pulmonary complications are frequent causes of increased blood lactate levels [9] and VA-ECMO should not be discontinued until resolution of pulmonary failure. The same may apply to other severe conditions underlying hyperlactatemia. Since a mild increase in arterial lactate has been shown to increase the risk of death in critically ill patients [10], achieving a normal level of lactate before discontinuation of cardiopulmonary support therapies is a reasonable goal as shown in other subsets of critically ill patients [11].

Arterial lactate before starting VA-ECMO is widely recognized as a predictor of poor outcome [2,3,4,5,6,7], still, to the best of our knowledge, no data exists on the prognostic value of arterial lactate at the time of weaning from VA-ECMO. Similarly, the clearance of arterial lactate, even when adjusted for the duration of this salvage therapy, was also not associated with an increased risk of early mortality. The reason for this resides in the complexity of lactate metabolism and the heterogeneity of causes underlying its increased production and/or decreased removal [12,13].

## 5. Limitations

The retrospective nature is a limitation of the present study. Secondly, arterial lactate levels were measured using different laboratory methods and this may introduce a significant bias, which we attempted to mitigate using multilevel mixed-effects regression analysis. Thirdly, arterial lactate levels might have been affected by patients’ conditions and treatment methods, such as renal replacement therapy, use of certain drugs, poor nutritional status, and therapies for acid-base abnormalities [12,13]. In such cases, the level of arterial lactate does not reflect the metabolic state of the patient at weaning from VA-ECMO.

## 6. Conclusions

This multicenter study showed that increases in arterial lactate at the time of weaning from postcardiotomy VA-ECMO are associated with an increased risk of hospital mortality. Further studies are needed to confirm these findings and to evaluate whether arterial lactate may be a useful biomarker to guide the optimal timing of VA-ECMO weaning.

## Figures and Tables

**Table 1:** Patients’ data and predictors of hospital mortality after weaning from postcardiotomy venoarterial extracorporeal membrane oxygenation.

	Overall Series 388 Patients	Survivors 221 Patients	Deaths 117 Patients	Univariate Analysis *p*-Value	Univariate Analysis OR, 95%CI	Mixed-Effects Multivariate Analysis OR, 95%CI
**Baseline covariates**						
Age (years)	61.1 ± 13.3	59.5 ± 13.5	64.1 ± 12.6	0.001	1.029, 1.010–1.049	1.058, 1.030–1.086
Female gender	108 (32.0)	62 (27.9)	46 (39.7)	0.028	1.696, 1.056–2.723	
eGFR (mL/min/1.73 m^2^)	69 ± 29	71 ± 30	64 ± 27	0.013	0.991, 0.982–0.999	
Dialysis	11 (3.3)	7 (3.2)	4 (3.5)	1.000	1.092, 0.313–3.809	
Anemia	154 (45.6)	94 (42.3)	60 (51.7)	0.100	1.459, 0.929–2.291	
Diabetes	88 (26.0)	58 (26.1)	30 (25.9)	0.958	0.986, 0.591–1.646	
Recent myocardial infarction	73 (21.6)	47 (21.2)	26 (22.3)	0.792	1.076, 0.625–1.850	
Prior stroke	23 (6.8)	16 (7.2)	7 (6.0)	0.684	0.827, 0.330–2.071	
Atrial fibrillation	80 (23.7)	50 (22.5)	30 (25.9)	0.493	1.200, 0.712–2.021	
Pulmonary disease	41 (12.1)	24 (10.8)	17 (14.7)	0.304	1.417, 0.727–2.759	
Extracardiac arteriopathy	42 (12.4)	24 (10.8)	18 (15.5)	0.213	1.515, 0.785–2.924	
Active endocarditis	38 (11.2)	29 (13.1)	9 (7.8)	0.143	0.560, 0.256–1.226	
Prior cardiac surgery	79 (23.4)	45 (20.3)	34 (29.3)	0.062	1.631, 0.973–2.734	2.022, 1.063–3.845
Coronary artery disease	136 (40.2)	91 (41.0)	45 (39.8)	0.696	0.912, 0.576–1.444	
Left ventricular ejection fraction ≤50%	203 (60.4)	130 (58.8)	73 (63.5)	0.336	1.217, 0.765–1.936	
Critical preoperative state	119 (35.2)	74 (33.3)	45 (38.8)	0.318	1.268, 0.795–2.020	
Ventricular arrhythmia	19 (5.6)	7 (3.2)	12 (10.3)	0.006	3.544, 1.355–9.266	5.587, 1.604–19.462
Preoperative IABP	29 (8.6)	17 (7.7)	12 (10.3)	0.402	1.391, 0.641–3.022	
Stroke/unconsciousness	9 (2.7)	3 (1.8)	5 (4.3)	0.174	2.455, 0.646–9.324	
Emergency procedure	81 (24.01)	57 (25.7)	24 (20.7)	0.308	0.755, 0.440–1.297	
PC-ECMO score	3.4 ± 2.2	3.0 ± 2.1	4.1 ± 2.4	<0.0001	1.230, 1.108–1.364	
EuroSCORE II (%)	14.2 ± 16.5	13.0 ± 15.4	16.3 ± 18.2	0.051	1.012, 0.98–1.025	
**Operative data**						
Any CABG	153 (45.3)	97 (43.7)	56 (48.3)	0.422	1.203, 0.767–1.887	
Aortic valve replacement	100 (29.6)	66 (29.7)	34 (29.3)	0.936	0.980, 0.599–1.604	
Aortic valve repair	2 (0.6)	2 (0.9)	0 (0)	0.305	-	
Mitral valve replacement	85 (25.1)	55 (24.8)	30 (25.9)	0.827	1.059, 0.633–1.773	
Mitral valve repair	51 (15.1)	35 (15.8)	16 (13.8)	0.630	0.855, 0.451–1.620	
Tricuspid valve replacement	9 (2.7)	6 (2.7)	3 (2.6)	1.000	0.956, 0.235–3.893	
Tricuspid valve repair	35 (10.4)	23 (10.4)	12 (10.3)	0.996	0.998, 0.478–2.086	
Aortic surgery	56 (16.6)	43 (19.4)	13 (11.2)	0.055	0.525, 0.270–1.023	
Aortic arch replacement	7 (2.1)	5 (2.3)	2 (1.7)	1.000	0.761, 0.145–3.986	
Surgery for GUCH disease	8 (2.4)	2 (0.9)	6 (5.2)	0.022	6.000, 1.191–30.215	22.910, 3.024–173.525
Repair of septum or ventricle	10 (3.0)	6 (2.7)	4 (3.4)	0.741	1.286, 0.355–4.650	
Pulmonary thromboendarterectomy	6 (1.8)	4 (1.8)	2 (1.7)	1.000	0.956, 0.173–5.299	
Other major cardiac surgery	8 (2.4)	3 (1.4)	5 (4.3)	0.129	3.288, 0.772–14.011	
Aortic cross-clamp time (min)	125 ± 74	126 ± 77	121 ± 69	0.899	0.999, 0.996–1.002	
.Cardiopulmonary bypass time (min)	219 ± 122	213 ± 118	230 ± 129	0.156	1.001, 0.999–1.003	
**VA-ECMO data**						
VA-ECMO immediately after surgery	207 (61.2)	135 (60.8)	72 (62.1)	0.822	1.055, 0.655–1.674	
Central cannulation	105 (31.1)	56 (25.2)	49 (42.2)	0.001	2.168, 1.346–3.493	
Arterial lactate at start of VA-ECMO (mmol/L)	6.1 ± 4.0	5.9 ± 4.0	6.3 ± 3.8	0.215	1.024, 0.968–1.083	
Arterial pH at start of VA-ECMO	7.31 ± 0.12	7.31 ± 0.11	7.30 ± 0.13	0.900	0.739, 0.109–4.982	
Arterial lactate at weaning from VA-ECMO (mmol/L)	1.9 ± 1.6	1.6 ± 1.2	2.4 ± 2.2	<0.0001	1.331, 1.140–1555.	1.426, 1.157–1.758
Arterial pH at weaning from VA-ECMO	7.34 ± 0.55	7.34 ± 0.56	7.33 ± 0.53	0.333	0.952, 0.641–1.413	
Duration of VA-ECMO (days)	8.1 ± 6.3	7.6 ± 5.9	8.9 ± 6.7	0.067	1.033, 0.997–1.070	1.063, 1.018–1.111

Continuous variables are reported as the mean ± standard deviation. Categorical variables are reported as counts and percentages. Anemia is defined as baseline hemoglobin concentration <12.0g/L in women and <13.0 g/L in men. OR, odds ratio; CI, confidence interval; eGFR, estimated glomerular filtration rate according to the Modification of Diet in Renal Disease equation; IABP, intra-aortic balloon pump; CABG, coronary artery bypass grafting; GUCH, grown-up congenital disease; EuroSCORE, European System for Cardiac Operative Risk Evaluation. Clinical variables are according to the EuroSCORE II definition criteria.

**Table 2:** Data of unmatched and propensity score-matched patients with arterial lactate at postcardiotomy VA-ECMO weaning less or higher than 1.4 mmol/L in the subgroup with normal lactate level or mild hyperlactatemia.

	Unmatched Patients			Propensity Score Matched Patients		
	Lactate <1.4 mmol/L 157 Patients	Lactate ≥1.4 mmol/L 104 patients	Standardized Differences	Lactate <1.4 mmol/L 87 Patients	Lactate ≥1.4 mmol/L 87 Patients	Standardized Differences
**Baseline covariates**						
Age (years)	61.2 ± 13.5	59.2 ± 12.9	0.154	60.6 ± 15.3	60.5 ± 12.7	0.013
Female gender	47 (29.9)	33 (31.7)	0.039	27 (31.0)	27 (31.0)	0.000
eGFR (mL/min/1.73 m^2^)	72 ± 31	67 ± 32	0.164	68±30	69±33	0.037
Dialysis	7 (4.5)	3 (2.9)	0.085	3 (3.4)	3 (3.4)	0.000
Anemia	67 (42.7)	52 (50.0)	0.147	45 (51.7)	40 (46.0)	0.115
Diabetes	42 (26.8)	30 (28.8)	0.047	26 (29.9)	26 (29.9)	0.000
Recent myocardial infarction	36 (22.9)	25 (24.0)	0.026	19 (21.8)	20 (23.0)	0.027
Prior stroke	7 (4.5)	10 (9.6)	0.202	7 (8.0)	7 (8.0)	0.000
Atrial fibrillation	30 (19.1)	25 (24.0)	0.120	18 (20.7)	20 (23.0)	0.056
Pulmonary disease	18 (11.5)	16 (15.4)	0.115	15 (17.2)	13 (14.9)	0.063
Extracardiac arteriopathy	16 (10.2)	13 (12.5)	0.073	11 (12.6)	11 (12.6)	0.000
Active endocarditis	16 (10.2)	18 (17.3)	0.208	14 (16.1)	11 (12.6)	0.098
Prior cardiac surgery	31 (19.7)	29 (27.9)	0.192	25 (28.7)	22 (25.3)	0.078
Coronary artery disease	68 (43.3)	41 (39.4)	0.079	38 (43.7)	36 (41.4)	0.047
Left ventricular ejection fraction ≤50%	96 (61.1)	65 (62.5)	0.028	52 (59.8)	56 (64.4)	0.095
Critical preoperative state	52 (33.1)	46 (44.2)	0.229	33 (37.9)	33 (37.9)	0.000
Ventricular arrhythmia	6 (3.8)	8 (7.7)	0.167	5 (5.7)	6 (6.9)	0.047
Preoperative IABP	14 (8.9)	10 (9.6)	0.024	7 (8.0)	8 (9.2)	0.040
Stroke/unconsciousness	2 (1.3)	5 (4.8)	0.207	2 (2.3)	1 (1.1)	0.088
Emergency procedure	35 (22.3)	33 (31.7)	0.214	24 (27.6)	21 (24.1)	0.079
PC-ECMO score	3.1±2.0	3.4±2.4	0.153	3.4±2.2	3.3±2.2	0.074
EuroSCORE II (%)	12.9±15.3	16.2±17.6	0.199	15.3±16.6	14.6±17.4	0.042
**Operative data**						
Any CABG	77 (49.0)	43 (41.3)	0.155	42 (48.3)	38 (43.7)	0.092
Aortic valve replacement	44 (28.0)	34 (32.7)	0.102	24 (27.6)	25 (28.7)	0.26
Aortic valve repair	1 (0.6)	0	0.113	0	0	-
Mitral valve replacement	40 (25.5)	25 (24.0)	0.033	26 (29.9)	22 (25.3)	0.103
Mitral valve repair	16 (10.2)	17 (16.3)	0.182	8 (9.2)	15 (17.2)	0.239
Tricuspid valve replacement	5 (3.2)	1 (1.0)	0.157	0	1 (1.1)	0.152
Tricuspid valve repair	18 (11.5)	8 (7.7)	0.128	14 (16.1)	6 (6.9)	0.291
Aortic surgery	27 (17.2)	14 (13.5)	0.104	12 (13.8)	10 (11.5)	0.069
Aortic arch replacement	1 (0.6)	2 (1.9)	0.115	0	2 (2.3)	0.261
Surgery for GUCH disease	3 (1.9)	4 (3.8)	0.116	3 (3.4)	4 (4.6)	0.059
Repair of septum or ventricle	3 (1.9)	4 (3.8)	0.116	3 (3.4)	2 (2.3)	0.069
Pulmonary thromboendarterectomy	2 (1.3)	3 (2.9)	0.113	2 (2.3)	3 (3.4)	0.069
Other major cardiac surgery	4 (2.5)	2 (1.9)	0.042	2 (2.3)	2 (2.3)	0.000
Aortic cross-clamp time (min)	120 ± 71	121 ± 79	0.022	120 ± 70	119 ± 78	0.002
Cardiopulmonary bypass time (min)	212 ± 115	211 ± 131	0.088	209 ± 106	206 ± 127	0.028
**VA-ECMO data**						
VA-ECMO immediately after surgery	94 (59.9)	59 (56.7)	0.064	51 (58.6)	47 (54.0)	0.093
Central cannulation	40 (25.5)	32 (30.8)	0.118	24 (27.6)	27 (31.0)	0.076
Arterial lactate at start of VA-ECMO (mmol/L)	5.4 ± 3.8	6.8 ± 4.2	0.337	6.1 ± 4.0	6.2 ± 3.8	0.019
Arterial pH at start of VA-ECMO	7.31 ± 0.12	7.30 ± 0.12	0.095	7.29 ± 0.11	7.30 ± 0.12	0.055
Arterial lactate at weaning from VA-ECMO (mmol/L)	1.0 ± 0.3	1.7 ± 0.2	-	1.0 ± 0.3	1.7 ± 0.2	-
Arterial pH at weaning from VA-ECMO	7.40 ± 0.22	7.34 ± 0.58	-	7.41 ± 0.6	7.38 ± 0.33	-
Duration of VA-ECMO (days)	8.4 ± 6.5	8.9 ± 6.5	0.088	8.5 ± 6.8	9.0 ± 6.6	0.067

Continuous variables are reported as the mean ± standard deviation. Categorical variables are reported as counts and percentages. Anemia is defined as baseline hemoglobin concentration <12.0g/L in women and <13.0 g/L in men. eGFR, estimated glomerular filtration rate according to the Modification of Diet in Renal Disease equation; IABP, intra-aortic balloon pump; CABG, coronary artery bypass grafting; GUCH, grown-up congenital disease; EuroSCORE, European System for Cardiac Operative Risk Evaluation. Clinical variables are according to the EuroSCORE II definition criteria.
